# The Effect of Work Environment and Work‐Related Characteristics on Perceived Individual Work Performance of Front‐Line Nurse Managers: A Cross‐Sectional Study

**DOI:** 10.1002/nur.22466

**Published:** 2025-04-14

**Authors:** Tuğba Yeşilyurt Sevim, Emine Türkmen, Nilgün Göktepe, Nuray Erdemir Çakmak, Eda Özlem Ünal, Esra Adigüzel, Ramiz İşçi

**Affiliations:** ^1^ Nursing Department Istinye University Faculty of Health Sciences Istanbul Turkey; ^2^ Nursing Department Ordu University Faculty of Health Sciences Ordu Turkey; ^3^ MLPCARE Patient Care Services Istanbul Turkey; ^4^ Natural Clinic Istanbul Turkey

**Keywords:** front‐line nurse manager, individual work performance, nurse manager, work environment

## Abstract

This study aimed to examine the effect of work environment and work‐related characteristics of front‐line nurse managers on their perceived individual work performance. It was conducted between March and May of 2022 with 219 front‐line nurse managers working in a private chain hospital group in Türkiye. The Nurse Manager Practice Environment Scale and the Individual Work Performance Questionnaire were used for data collection. Descriptive statistics, Spearman correlation analysis, and generalized linear model regression analysis were used in data analysis. A positive correlation was found between the scores obtained from the two scales (*r* = 0.453; *p* < 0.001). Regression analysis revealed that two independent variables—nurse manager's work environment and duration of managerial experience—predicted nurse managers' perceived individual work performance level, with these variables explaining 28.1% of the total variance. Results showed that a favorable work environment and four or more years of managerial experience enhanced the perceived individual performance of front‐line nurse managers. These findings suggest that to improve the individual work performance of front‐line nurse managers, their work environment must also be improved. In addition, retention strategies that target experienced front‐line nurse managers are critical to maintaining high levels of performance.

## 1 Introduction

In today's complex and rapidly changing healthcare environments, the leadership role of nurse managers significantly impacts the achievement of patient outcomes (e.g., quality of care, patient safety) (Warshawsky et al. 2022; Grandfield et al. 2023), nurse outcomes (e.g., job satisfaction, retention) (Cummings et al. 2018; Grandfield et al. 2023; Zaghini et al. 2020), and organizational outcomes (e.g., staff turnover, financial performance) (Cummings et al. 2018; Hult et al. 2023; Suliman et al. 2020). Concurrently, such outcomes also reflect nurse managers' work performance (Warshawsky 2020). In today's world in which value‐based healthcare is at the forefront, nurse managers are responsible for practices such as providing quality, safe, effective, fair, and efficient healthcare; responding to the expectations of patients and their relatives; ensuring continuity of care; and reducing costs (American Organization of Nurse Executives AONE, American Organization for Nursing Leadership AONL 2022). Front‐line nurse managers (FLNMs) have a significant impact on the success of the organization because they play a key role in day‐to‐day operations of the healthcare unit and work in close contact with patients, families, and other members of the healthcare team (Goktepe et al. 2018). In addition to playing a critical role in the lives of staff nurses and in the achievement of quality care outcomes, FLNMs are expected to develop their leadership/managerial skills and integrate these skills with clinical and managerial processes (Goktepe et al. 2018; Gunawan et al. 2023). By improving their own performance, FLNMs may empower staff nurses, provide direction, increase performance levels, and thus achieve quality (patient and nurse) outcomes (Hult et al. 2023; Nurmeksela et al. 2021).

Considering the effect of nurse managers' performance on outputs, the factors affecting their performance, as well as antecedents of their performance, must be examined (Batson and Yoder 2012). A concept analysis study by Gunawan and Aungsuroch (2017) on the managerial competence of FLNMs revealed that when nurse managers are motivated and satisfied with their jobs, they enhance their competencies by developing skills in leadership, planning, resource management, and team organization. Consequently, this leads to improved care outcomes through increased patient and staff satisfaction (Gunawan and Aungsuroch 2017). Previous studies have emphasized the importance of examining the effectiveness of nurse managers' managerial skills and leadership behaviors, as well as the quality of their work environment (Chen et al. 2024; Grandfield et al. 2023; Warshawsky 2020). However, the subject of FLNMs' work environments has not yet been sufficiently studied. Most previous studies have focused primarily on the work environments of staff nurses (Lake et al. 2019; Dutra and Guirardello 2021; Sarıköse and Göktepe 2022), and only a few studies have evaluated the work environment from the perspective of nurse managers, finding significant associations between work environment and various outcomes. These include nurse and nurse manager outcomes such as turnover, job satisfaction (Grandfield et al. 2023), burnout (Miller et al. 2023; Takemura et al. 2020), organizational silence (Aslan et al. 2022), and joy in work (Grandfield et al. 2023), as well as patient outcomes such as missed care and quality of care (Grandfield et al. 2023). Furthermore, nurse managers' work environment has been identified as a critical driver of performance (Grandfield et al. 2023). The literature reveals that although a limited number of studies have examined FLNM work environment outcomes as indicators of work performance (Cummings et al. 2018; Warshawsky 2020), no study to our knowledge has directly examined the relationship between nurse managers' work environment and perceived work performance. On this point, Bandura (1997) self‐efficacy theory provides an important theoretical framework for explaining how perceived performance is shaped at both the individual and organizational levels. According to this theory, an individual's perception of their own abilities (self‐efficacy) can directly influence their actual performance. In other words, the self‐efficacy one has in relation to a specific task can directly impact their perception of performance. When an individual perceives themselves as successful, they are more likely to exert greater effort, which can lead to improved performance (Bandura 1997; Iroegbu 2015). Therefore, this study contributes to the literature by revealing the antecedents of FLNM performance and examining the effect of FLNMs' work environment on the perceived individual performance of FLNMs.

### 1.1 Background

#### 1.1.1 Nurse Manager Work Environment

The nurse manager work environment is defined as an organizational context that affects the skills and competencies of nurses (Warshawsky et al. 2013a). Organizational context affects the work results/performance of employees (Warshawsky 2020). As the nurse manager is also an employee, her/his relations with executive managers and managerial support are extremely important. Moreover, such support is reflected in nurse managers' work performance and relationships with other health team members and staff nurses.

A review of the literature identified some key studies that define the working environment of nurse managers. Warshawsky et al. (2013a, 2013b) highlighted eight critical areas that influence nurse managers' practice: empowering administrative leaders to create a culture of patient safety, constructive nurse manager–director relationships, culture of generativity, adequate budgeted resources, culture of meaning, collegial nurse manager‐physician relationships, effective nurse manager‐unit staff relationships, and fair and manageable workload. In a study conducted by Flynn et al. (2016), nurse managers described key characteristics that create a work environment supportive of their managerial practice; these characteristics included collegial supervisory partnerships, entrusted authority, adequate resources, knowledge sharing, peer collaboration, organizational commitment to quality, and safety. In addition, Huddleston and Gray (2016) examined both staff nurses' and nurse managers' perceptions of a healthy work environment, highlighting the importance of appropriate staffing, authentic leadership, effective decision‐making, meaningful recognition, skilled communication, true collaboration, genuine teamwork, and physical and psychological safety. These studies highlight the importance of empowered leadership, sufficient resources, effective communication, and a safe working environment in creating conditions conducive to nurse managers' successful leadership.

#### 1.1.2 Nurse Manager Work Performance

Defined as an employee's contribution to organizational goals (Aslan and Yıldırım 2017), level of work completed, or type of behavior, performance is generally considered in two dimensions: task performance and contextual performance (Borman and Motowidlo 1993). While task performance refers to the achievement of the objectives found in job descriptions, contextual performance refers to additional constructive behaviors that positively contribute to the achievement of organizational goals (Aslan and Yıldırım 2017; Koopmans et al. 2013). Organizations should focus not only on task performance but also on contextual performance, as employee performance is a motivational and social process shaped by interactions between employees and organizational factors through managerial interventions (Rusu et al. 2016).

As formal leaders of the institution/organization in which they work, nurse managers support the front‐line healthcare team (Aslan et al. 2022), and the outcome of their practice is known as work performance (Warshawsky 2020). The work performance of nurse managers reflects their effectiveness and is assessed through organizational, staff, and patient outcomes (Schlotzhauer et al. 2023; Warshawsky 2020). Management practice is a broad concept that reflects how nurse managers fulfill their responsibilities and roles specified in their job descriptions. Although the basic job descriptions for nurse managers are generally similar across organizations or departments within an organization, there are differences in how individual nurse managers fulfill their roles.

Complexity science's and complexity leadership theory's basic tenet that organizations are evolving and adaptive systems may contribute to explaining the practice or performance of nurse managers (Uhl‐Bien et al. 2020). Given the complexity of the organizational context in healthcare institutions, nurse managers act as a buffer against forces external to the unit (Cummings et al. 2018). This buffering process is two‐way. The first part involves creating a professional work environment that builds accountability and commitment to quality patient care and outcomes for nurses and other members of the healthcare team (Van Bogaert et al. 2015), while the second involves communicating the strategic plan and vision to nurses in terms of administrative processes. Both require effective communication and cooperation with both employees and executive managers (Gutberg and Berta 2017). In this context, nurse managers may contribute to their own development as well as that of their teams by honing their awareness of their own tasks and contextual performances through self‐assessment, as well as by knowing what affects these performances in the context of the characteristics of the work environment.

## 2 Methods

### 2.1 Aim

This study was conducted to examine the effect of work environment and job‐related characteristics of FLNMs on their perceived individual work performance.

### 2.2 Design

This study used a descriptive cross‐sectional design.

### 2.3 Sample and Settings

The population of this study consisted of 300 FLNMs working in 30 hospitals in a private chain hospital group in Türkiye. The online survey link was distributed to all FLNMs via executive nurses. A total of 225 responses were collected, and six incomplete surveys were excluded, resulting in data collected from 219 front‐line nurse managers. The adequacy of the sample size (*n* = 219) was verified by post hoc power analysis using G*Power software (version 3.1.9.7, Kiel, Germany). For the effect of 10 independent variables on the dependent variable (individual work performance) with an *R*² of 0.281, the effect size was calculated as *f*² = 0.3908. At the 95% confidence level (1‐α), the power of the study was found to be greater than 99%.

### 2.4 Measurements

The “Demographic and Work‐Related Variables Questionnaire,” the “Nurse Manager Practice Environment Scale,” and the “Individual Work Performance Questionnaire” were used to collect the research data.

#### 2.4.1 Demographic and Work‐Related Variables Questionnaire

This form, developed by the authors, consists of 15 questions including the demographic and work‐related characteristics of nurses.

#### 2.4.2 Nurse Manager Practice Environment Scale

The NMPES was developed by Warshawsky et al. in 2013 and adapted into Turkish by Tosun and Yıldırım (2021b). It consists of 44 items and eight subscales (empowering administrative leaders to create a culture of patient safety, constructive NM‐director relationships, culture of generativity, adequate budgeted resources, culture of meaning, collegial relationships between NMs and physicians, effective NM‐staff relationships, and fair and manageable workload). Items were scored on a Likert‐type scale ranging from one to six. The scale and its sub‐dimensions are evaluated on an average score, with scores approaching six indicating more positive perceptions of the work environment. Cronbach's alpha value for the overall scale was 0.97 in the original study (Warshawsky et al. 2013b), 0.96 in the Turkish adaptation study (Tosun and Yıldırım 2021b), and 0.98 in the current study.

#### 2.4.3 Individual Work Performance Questionnaire (IWPQ)

This questionnaire was developed by Koopmans et al. (2013) and adapted into Turkish by Köroğlu et al. (2021). The 5‐point Likert‐type scale consists of 18 items and three sub‐dimensions. In the Turkish adaptation study, it was reduced to 14 items containing three sub‐dimensions (Task performance, Contextual performance, and Counterproductive work behavior). Items 12–14 in the “Counterproductive work behavior” dimension are reverse scored. Scale scoring is calculated by dividing the total scale score by the number of items. A high score on the scale indicates a perception of increased work performance. In the original form of the scale, Cronbach's alpha value was 0.78 for “Task performance,” 0.85 for “Contextual performance,” and 0.79 for “Counterproductive work behavior” (Koopmans et al. 2013, 2016). In the Turkish adaptation study, Cronbach's alpha was found to be 0.80 for the overall scale and 0.86, 0.78, and 0.72 for the sub‐dimensions, respectively (Köroğlu et al. 2021). In the current study, Cronbach's alpha value was 0.86 for the overall scale and 0.90, 0.87, and 0.90 for the sub‐dimensions, respectively.

### 2.5 Data Collection

Data were collected between March and May of 2022. The link to the online survey was shared by the executive nurses of each hospital in their nurse managers' WhatsApp groups. The questionnaire was preceded by informational text explaining the purpose and scope of the study, indicating that participation was voluntary and anonymous (containing no personal information about participants). The survey was designed to exclude any personal information (e.g., name, email address) and was configured to ensure that the survey platform did not record IP addresses or device identifiers, guaranteeing that participants' responses were completely anonymous. Unique links were not used to prevent individual tracking of responses, and the survey link was shared publicly.

Participants who consented to participate in the study (by ticking the relevant box) were given access to the survey questions. The survey link, including a reminder message, was re‐shared three times in WhatsApp groups at 15‐day intervals, and the survey took ~15 min to complete.

### 2.6 Ethical Considerations

Ethics committee approval (XX) for this study was obtained from a university, and approval was also obtained from the headquarters of the relevant hospitals in which the study was conducted.

### 2.7 Data Analysis

Data analysis was performed using IBM SPSS Statistics 27.0 (IBM Corp., Armonk, NY, USA). In the study, the normality assumption of the NMPES and IWPQ scores was tested using the Kolmogorov‐Smirnov test for skewness and kurtosis values. The Kolmogorov‐Smirnov test value was 0.101 (*p* < 0.001) for NMPES and 0.086 (*p* < 0.001) for IWPQ. The skewness value for NMPES was −1.08 (SE = 0.16), and the kurtosis value was 2.46 (SE = 0.33). The skewness value for IWPQ was −0.77 (SE = 0.16), and the kurtosis value was 0.36 (SE = 0.33). Since the *p*‐value of the Kolmogorov‐Smirnov test was significant and the Kurtosis value of NMPES was not within the desired range of values (−2 to +2), it was determined that the data were not normally distributed (Kim 2013; Mishra et al. 2019). Categorical variables were presented as frequency (*n*, %), and continuous variables were presented as mean, standard deviation, median, and interquartile range (IQR). Cronbach's alpha reliability coefficients were calculated to determine the reliability of the scales used in the study. The Mann‐Whitney *U* test was used for comparisons between two groups to determine the difference between IWPQ scores according to independent variables. The Kruskal‐Wallis test was used for comparisons between more than two groups. The level of association between NMPES and IWPQ scores was examined using Spearman correlation analysis. Generalized linear model (GLM) regression analysis was used to determine the effect of the independent variables on the dependent variable (individual work performance). GLM analysis was preferred because the scale scores were not normally distributed. Considering the basic analyses for the dependent variable, ten independent variables with a 10% level of statistical significance were selected. These were: (1) categorical variables—educational background, years of management experience, scheduling, daily working hours, general job satisfaction, satisfaction with career opportunities at the hospital, satisfaction with quality of care at the hospital, satisfaction with patient safety practices at the hospital, and satisfaction with salary, and (2) a numeric variable—the NM Work Environment Scale. The results for the regression model were evaluated at a 95% confidence interval, and significance was assessed at *p* < 0.05 (two sided).

## 3 Results

Nurses' demographics and work‐related variables are shown in Table 1. The mean age of the participants was 31.8 years (SD = 8.7), the mean total duration of professional experience was 11.7 years (SD = 8.1), and the mean years of management experience was 4.2 years (SD = 4.3). Almost three‐fourths (71.2%) of the participants worked day shifts, 67.6% worked 10 h or less per day, and 73.5% worked 50 h or less per week. More than half (56.2%) of the managers had participated in a nursing management education program, 83.6% were satisfied or very satisfied with career opportunities in the hospital, 90% were satisfied with the quality of care provided, 95% were satisfied with patient safety practices, and 55.7% were dissatisfied or very dissatisfied with the salary they received.

**Table 1:** Nurse manager's demographics and work‐related variables (*N* = 219).

Variables	*n* (%)	Mean (SD)
**Age (years)**		31.8 (8.7)
≤ 30 years	120 (54.8)	
> 30 years	99 (45.2)	
**Gender**		
Woman	176 (80.4)	
Man	43 (19.6)	
**Educational background**		
High school	117 (53.4)	
Associate degree	45 (20.5)	
Bachelor's degree	38 (17.4)	
Master's degree	19 (8.7)	
**Total professional experience**		11.7 (8.1)
≤ 11 years	123 (56.2)	
> 11 years	96 (43.8)	
**Employment duration at current hospital**		5.8 (4.6)
≤ 5 years	122 (55.7)	
> 5 years	97 (44.3)	
**Years of management experience**		4.2 (4.3)
≤ 4 years	153 (69.9)	
> 4 years	66 (30.1)	
**Scheduling**		
Day shift	156 (71.2)	
Shiftwork	63 (28.8)	
**Daily working hours**		
≤ 10 h	148 (67.6)	
> 10 h	71 (32.4)	
**Weekly working hours**		
≤ 50 h	161 (73.5)	
> 50 h	58 (26.5)	
**Nursing management education program**		
Participated	122 (56.2)	
Did not participate	97 (43.8)	
**General job satisfaction**		
Satisfied/Very satisfied	195 (89.0)	
Dissatisfied/Very dissatisfied	24 (11.0)	
**Satisfaction with career opportunities at hospital**		
Satisfied/Very satisfied	183 (83.6)	
Dissatisfied/Very dissatisfied	36 (16.4)	
**Satisfaction with nursing care quality at hospital**		
Satisfied/Very satisfied	197 (90.0)	
Dissatisfied/Very dissatisfied	22 (10.0)	
**Satisfaction with patient safety practices at hospital**		
Satisfied/Very satisfied	208 (95.0)	
Dissatisfied/Very dissatisfied	11 (5.0)	
**Satisfaction with salary**		
Satisfied/Very satisfied	97 (44.3)	
Dissatisfied/Very dissatisfied	122 (55.7)	

Abbreviation: SD, standard deviation.

NMPES and IWPQ scores are shown in Table 2. The mean score of the NMPES obtained from the FLNMs' self‐report was 4.94 (SD = 0.74). The highest mean score from its sub‐dimensions was 5.38 (SD = 0.73) for NMPES F8 (Effective NM‐Unit Staff Relationships), while the lowest mean score was 4.28 (SD = 1.11) for NMPES F4 (Adequate Budgeted Resources). The mean score for IWPQ was 4.24 (SD = 0.54). The highest mean score of the IWPQ sub‐dimensions was 4.06 (SD = 1.26) for IWPQ F3 (Counterproductive Work Behavior), with the scores of the remaining two sub‐dimensions being very similar—4.30 (SD = 0.67) for IWPQ F2 (Contextual Performance) and 4.29 (SD = 0.72) for IWPQ F1 (Task Performance).

**Table 2:** Descriptive statistics: Nurse Manager Work Environment Scale & Individual Work Performance Questionnaire scores (*N* = 219).

NM Work Environment Scale (NMWES)	Median (range)	$\bar{x}$ (SD)	Cronbach's Alpha
Overall scores (44 items)	4.9 (1.7–6)	4.94 (0.74)	0.976
Subscales			
NMWES F1. Empowering administrative leaders to create a culture of patient safety (15 items)	5 (1.5–6)	4.96 (0.78)	0.942
NMWES F2. Culture of meaning (4 items)	5 (1–6)	5.06 (0.82)	0.876
NMWES F3. Culture of generativity (6 items)	5 (1–6)	4.91 (0.83)	0.846
NMWES F4. Adequate budgeted resources (4 items)	4.2 (1–6)	4.28 (1.11)	0.741
NMWES F5. Fair and manageable workload (3 items)	5 (1–6)	4.65 (1.02)	0.707
NMWES F6. Constructive NM‐director relationships (6 items)	5.1 (1–6)	5.08 (0.87)	0.919
NMWES F7. Collegial relationships between NMs and physicians (3 items)	5.3 (2–6)	5.20 (0.74)	0.775
NMWES F8. Effective NM‐unit staff relationships (3 items)	5.6 (1–6)	5.38 (0.73)	0.880
**Individual Work Performance Questionnaire**			
Overall scores (14 items)	4.2 (2.4–5)	4.24 (0.54)	0.857
Subscales			
IWPQ F1. Task performance (5 items)	4.4 (1.8–5)	4.29 (0.72)	0.899
IWPQ F2. Contextual performance (6 items)	4.3 (2–5)	4.30 (0.67)	0.868
IWPQ F3. Counterproductive work behavior (3 items)	4.6 (1–5)	4.06 (1.26)	0.901

Abbreviations: NM, nurse manager; SD, standard deviation.

The results of Spearman correlation analyses showing the relationship between both scales and their subscales are presented in Table 3. The correlation coefficient between the two scale scores was 0.453, and the relationship between them was significant (< 0.001). In terms of correlations, the first two subscales of the IWPQ—Task Performance and Contextual Performance—were significantly correlated with each of the NMPES domains (*r*: 0.238–0.536, *p* < 0.001), and the third subscale of the IWPQ—Counterproductive Work Behavior, was correlated with only three NMPES subscales. These were: NMPES F2: Culture of Meaning (*r*: 0188, *p* < 0.01), NMPES F6: Constructive NM‐Director Relationships (*r*: 0169, *p* < 0.05), and NMPES F7: Collegial Relationships Between NMS and Physicians (*r*: 0140, *p* < 0.05).

**Table 3:** Relationship between Nurse Manager Work Environment Scale and Individual Work Performance Questionnaire (*N* = 219).

NM Work Environment Scale (No ♯ items)	Individual Work Performance Questionnaire							
	Overall scale (14 items)		F1: Task performance (5 items)		F2: Contextual performance (6 items)		F3: Counterproductive work behavior (3 items)	
	*r*	*p*	*r*	*p*	*r*	*p*	*r*	*p*
**Overall scale**	0.453	< 0.001	0.529	< 0.001	0.406	< 0.001	0.126	0.063
**Subscales**								
F1: Empowering administrative leaders to create a culture of patient safety	0.436	< 0.001	0.509	< 0.001	0.376	< 0.001	0.125	0.065
F2: Culture of meaning	0.473	< 0.001	0.490	< 0.001	0.435	< 0.001	0.188	0.005**
F3: Culture of generativity	0.481	< 0.001	0.532	< 0.001	0.481	< 0.001	0.109	0.108
F4: Adequate budgeted resources	0.277	< 0.001	0.447	< 0.001	0.248	< 0.001	0.017	0.800
F5: Fair and manageable workload	0.335	< 0.001	0.428	< 0.001	0.330	< 0.001	0.056	0.408
F6: Constructive NM‐director relationships	0.401	< 0.001	0.423	< 0.001	0.340	< 0.001	0.169	0.012*
F7: Collegial relationships between NMS and physicians	0.383	< 0.001	0.376	< 0.001	0.388	< 0.001	0.140	0.039*
F8: Effective –NM‐unit staff relationships	0.499	< 0.001	0.433	< 0.001	0.396	< 0.001	0.249	< 0.001

*r*: Spearman correlation analysis;

* *p* < 0.05

** *p* < 0.01.

The results of the comparison of the FLNMs' individual work performance scores according to demographic and work‐related factors are presented in Table 4. Generalized linear regression analysis was applied to determine the variables predicting the perceived individual work performance of FLNMs. In this analysis, two variables from 10 independent variables remained in the model. These variables were: overall NMPES and duration of management experience (>4 years). B value was 0.370 (SE = 0.055, *p* < 0.001) for NMPES and 0.177 (SE = 0.073, *p* < 0.05) for duration of management experience. These variables explained 28.1% of the total variance (*R* ^2^ = *0.281)* (Table 5).

**Table 4:** Individual performance scores of nurse managers according to their descriptive characteristics.

		IWP Score		
Variables	*n*	Median (IQR)	Test value	*p* value
**Age**			−0.264^a^	0.792
≤30 years	120	4.3 (4–4.6)		
>30 years	99	4.3 (3.9–4.6)		
**Gender**			–0.293^a^	0.769
Woman	176	4.3 (4–4.6)		
Man	43	4.2 (4.1–4.7)		
**Educational background**			6.736^b^	0.081*
High school	117	4.3 (4.1–4.7)		
Associate degree	45	4.4 (4.1–4.8)		
Bachelor's degree	38	4.1 (3.7–4.7)		
Master's degree	19	4.1 (3.8–4.4)		
**Total professional experience**			–0.339^a^	0.735
≤11 years	123	4.2 (4–4.6)		
>11 years	96	4.3 (3.9–4.7)		
**Employment duration at current hospital**			–0.039^a^	0.969
≤5 years	122	4.3 (4–4.6)		
>5 years	97	4.3 (4–4.7)		
**Years of management experience**			–1.776^a^	0.076*
≤4 years	153	4.2 (4–4.6)		
>4 years	66	4.4 (4–4.8)		
**Scheduling**			–1.811^a^	0.070*
Day shift	156	4.3 (4.1–4.7)		
Shiftwork	63	4.1 (3.8–4.6)		
**Daily working hours**			–2.012^a^	0.044**
≤10 h	148	4.4 (4.1–4.7)		
>10 h	71	4.1 (3.9–4.5)		
**Weekly working hours**			–0.598^a^	0.550
≤50 h	161	4.2 (4–4.7)		
>50 h	58	4.3 (3.8–4.6)		
**Nursing management education program**			–0.984^a^	0.325
Participated	122	4.3 (4.1–4.7)		
Did not participate	97	4.2 (3.9–4.5)		
**General job satisfaction**			–2.844^a^	0.004***
Satisfied/Very satisfied	195	4.3 (4–4.7)		
Dissatisfied/Very dissatisfied	24	4.1 (3.1–4.3)		
**Satisfaction with career opportunities at hospital**			–2.614^a^	0.009***
Satisfied/Very satisfied	183	4.3 (4.1–4.7)		
Dissatisfied/Very dissatisfied	36	4.1 (3.7–4.5)		
**Satisfaction with nursing care quality at hospital**			–2.969^a^	0.003***
Satisfied/Very satisfied	197	4.3 (4–4.7)		
Dissatisfied/Very dissatisfied	22	4.0 (3.6–4.3)		
**Satisfaction with patient safety practices at hospital**			–1.846^a^	0.065*
Satisfied/Very satisfied	208	4.3 (4–4.7)		
Dissatisfied/Very dissatisfied	11	4.1 (3.7–4.2)		
**Satisfaction with salary**			–2.260^a^	0.024**
Satisfied/Very satisfied	97	4.4 (4.1–4.8)		
Dissatisfied/Very dissatisfied	122	4.2 (3.9–4.5)		

^a^ Mann‐Whitney U test.

^b^ Kruskal‐Wallis test; IQR, Interquartile Range.

* *p* < 0.10

** *p* < 0.05

*** *p* < 0.01.

**Table 5:** Factors associated with individual performance of nurse managers *(Generalized Linear Model analysis results)*.

			95% CI			
Variables	*B*	SE	Lower	Upper	*z*	*P* value
**(Intercept)**	4.320	0.091	4.141	4.498	47.393	<0.001
**Years of management experience** *(> 4 y vs* ≤ *4 y*)**	0.177	0.073	0.033	0.321	2.411	**0.017** *
**Scheduling** *(daily shift vs night & daily shift*)**	0.047	0.117	−0.181	0.276	0.406	0.685
**Daily working hours** *(> 50 h vs* ≤ *50 h*)**	−0.011	0.109	−0.225	0.203	−0.104	0.917
**Level of education**	−0.059	0.033	−0.123	0.006	−1.792	0.075
**General job satisfaction** *(satisfied/very satisfied vs dissatisfied/very dissatisfied*)**	0.100	0.125	−0.145	0.344	0.800	0.425
**Satisfaction with career opportunities at hospital** *(satisfied/very satisfied vs dissatisfied/very dissatisfied*)**	−0.056	0.104	−0.260	0.148	−0.538	0.591
**Satisfaction with nursing care quality at hospital** *(satisfied/very satisfied vs dissatisfied/very dissatisfied*)**	−0.056	0.148	−0.347	0.235	−0.376	0.707
**Satisfaction with patient safety practices at hospital** *(satisfied/very satisfied vs dissatisfied/very dissatisfied*)**	−0.117	0.194	−0.497	0.263	−0.601	0.548
**Satisfaction with salary** *(satisfied/very satisfied vs dissatisfied/very dissatisfied*)**	−0.010	0.070	−0.146	0.126	−0.145	0.885
**NMPES**	0.370	0.055	0.263	0.477	6.785	**<0.001** *

Abbreviation: NMPES, Nurse Manager Practice Environment Scale.

* *p* < 0.05.

** Reference value; SE, standard error; CI, Confidence interval; Model type, Linear; Link function, Identity; *R* ^2^: 0.281.

## 4 Discussion

In order for executive health managers to achieve desired performance in their organizations, they must create a positive work environment for the FLNMs with whom they work (Warshawsky et al. 2013b).

In this study, the mean score of the NMPES scale was found to be 4.94 (0.74) on a 6‐point Likert scale (Table 2), indicating that the participating FLNMs perceived their work environment positively (Warshawsky et al. 2013). Similarly, both international studies (Chen et al. 2024; Warshawsky et al. 2016) and studies conducted in Türkiye (Aslan et al. 2022; Tosun and Yıldırım 2021a) reported that nurse managers rated their work environment positively. The positive ratings found in the current study may be due to the fact that the institutions in which the research was conducted were private and accredited hospitals, which are likely to provide more favorable working conditions for nurse managers compared to public healthcare settings in Türkiye. Likewise, Göktepe et al. (2020) found that management processes were more structured in accredited private hospitals. Nurse managers in these institutions reported more frequent participation in decision‐making processes, stronger representation, more robust institutional support mechanisms, and more favorable working conditions. In contrast, nurse managers working in public hospitals reported various challenges including low autonomy, limited involvement in decision‐making processes, and excessive administrative responsibilities. Similarly, Tosun and Yıldırım (2021a) found that nurse managers working in private hospitals rated their practice environment more positively than those working in Ministry of Health and university hospitals in Türkiye. This finding suggests that the work environment may differ within different sectors of healthcare in the country.

Among the sub‐dimensions of the NMPES, the highest score was observed in the “effective nurse manager‐unit staff relationships” sub‐dimension, while the lowest score was observed in the “adequate budgeted resources” sub‐dimension. The “effective nurse manager‐unit staff relationships” sub‐dimension refers to meaningful working relationships between FLNMs and staff that contribute to high‐quality patient outcomes (Warshawsky et al. 2013). Overall, this finding is consistent with results reported in previous international studies (Miller et al. 2023; Tarasenko et al. 2022; Warshawsky et al. 2016) and studies conducted in Türkiye (Aslan et al. 2022; Tosun and Yıldırım 2021a), indicating the importance of effective nurse manager‐staff relationships and highlighting cross‐country similarities regarding challenges related to budget constraints. Studies have shown that the quality of relationships between managers and staff is associated with improved nursing and unit outcomes. Relational leadership styles play a key role in fostering positive work environments, job satisfaction, and quality patient care by enhancing nurse managers' skills in critical thinking, conflict management, and team building (Cummings et al. 2018; Keith et al. 2021; McCauley et al. 2020; Nurmeksela et al. 2021). The “adequate budgeted resources” sub‐dimension received the lowest score on the scale. In the study by Warshawsky et al. (2013a), nurse managers indicated that they wanted administrative leaders to define organizational priorities and make budget decisions in line with the organization's mission, vision, and values. The same study highlighted that nurse managers felt that current economic conditions in healthcare required the efficient management of human and material resources but that budget constraints conflicted with providing the best and safest patient care, leading to burnout. The financial resources available to nurse managers are critical to the sustainability of nursing services and improvement of quality of patient care (Jäppinen et al. 2022). The findings of this study suggest that FLNMs face difficulties in accessing the necessary resources to carry out their roles. While structural and policy differences vary between countries, the need for global strategies designed to support nurse managers in securing adequate financial resources for sustainable care is clear.

This study found that the mean total score of the IWPQ was 4.24 ± 0.54 (Table 3), revealing that the FLNMs participating in the study exhibited a high level of perceived individual work performance (Köroğlu et al. 2021). While the literature contained no study measuring the perceived individual work performance of FLNMs, this finding suggests that despite the shortage of resources and increased workloads in institutions, nurse managers are motivated to provide uninterrupted service and coordinate the components of such service, either directly or indirectly.

This study found that the duration of managerial experience positively affected FLNMs' perception of individual work performance, with those having more than 4 years of total working experience reporting higher perceived work performance. Findings from international studies support this relationship, indicating that the perceived competence level of nurse managers was directly proportional to their experience, and revealing that competence is a behavior that results in high performance (Grandfield et al. 2023; Ochonma et al. 2018; Warshawsky et al. 2022). One study even showed that experience had twice the impact on nurse managers' perceived competence as postgraduate leadership training (Warshawsky et al. 2020). A recent study found that the highest‐performing nurse managers in the sample had spent more years in nursing, more years as a nurse manager, and more years in their current role than the lowest performers (Schlotzhauer et al. 2023). Similarly, a study of senior nurse managers found that those with less than 6 years of experience had lower self‐perceived managerial performance and required more training in leadership, supervision, staff and resource management, and planning and innovation (Tsang 2017). Studies reported that less experienced nurse managers had the most difficulty in saying no to tasks, in making changes to their workload, and in adopting effective coping strategies, while experienced managers contributed more to the organization by reducing the negative effects of challenging workplace experiences (Grandfield et al. 2023). The results obtained in this study suggest that the management skills acquired over the years, as well as the competencies gained, improve the work performance of FLNMs. While no study in Türkiye has directly examined the relationship between nurse managers' managerial experience and performance, national research sheds light on the impact of experience on leadership competencies and managerial skills. One study found that first‐level nurse managers with more than 5 years of managerial experience had significantly higher self‐perceived competence scores (Goktepe et al. 2018). Both international and national studies highlight the role of managerial experience in shaping nurse managers' competencies, and the findings from Türkiye support international evidence that experience is a key factor in effective nursing leadership.

Results of the current study showed that a favorable work environment, as measured by NMPES, is associated with the perceived individual work performance of FLNMs. A review of the literature revealed that while there were no studies examining the direct relationship between FLNMs' work environment and perceived work performance, some studies have examined practice environment outcomes as indicators of work performance (Cummings et al. 2018; Warshawsky 2020). These practice outcomes reflect the effectiveness of nurse managers and are assessed through organizational, staff, and patient outcomes (Hult et al. 2023; Grandfield et al. 2023; Schlotzhauer et al. 2023). According to previous studies, nurse managers report that practice environments with positive supportive cultures, relationships, and adequate resources support their work performance (Grandfield et al. 2023; Keith et al. 2021; Nurmeksela et al. 2021). In addition, Schlotzhauer et al. (2023) found that the highest‐performing nurse managers rated their practice environment more positively on all sub‐dimensions of the NMPES. Based on the results of the current study, nurse manager's work environment may be considered an important factor that influences perceived performance.

### 4.1 Limitations

Because this study is likely the first known study to examine the effect of work environment and work‐related characteristics of FLNMs on their perceived individual work performance, discussion of relevant findings is limited. In addition, the results of the study are based on a sample of 30 private hospitals, limiting the generalizability of the findings to other types of hospitals or healthcare facilities. These private hospitals may differ from public hospitals or other private institutions in terms of organizational structure, culture, and work environment, which may affect the applicability of the results. Further research is needed to determine the generalizability of these findings. Another limitation is that both performance and work environment measures were self‐reported, reflecting subjective perceptions rather than objective measures of work environment and work performance. In addition, because the data are cross‐sectional, we cannot draw causal inferences concerning the studied factors. Furthermore, the data were collected via an online form, potentially limiting its depth. Finally, as both predictor and outcome variables were reported by the same participants, there is the potential for same‐source bias, which could affect the validity of the findings.

## 5 Conclusion

This study's results showed that a favorable work environment and more than 4 years of managerial experience improved the perceived individual work performance of FLNMs. The findings underline the critical role of a supportive and empowering work environment in fostering high performance among FLNMs. Furthermore, the study emphasized that the competencies and skills gained through managerial experience positively influence the perception of performance.

## Implications for practice

This study's results suggest that to achieve better organizational, staff, and patient outcomes, healthcare organizations must create practice environments that support the strong work performance of nurse managers. Healthcare organizations should also prioritize investments in improving FLNMs' working conditions through the provision of adequate resources, development of supportive professional relationships, and the fostering of a culture of empowerment and productivity. Policy makers and organizational leaders should allocate resources and establish frameworks that support FLNMs' critical needs, ensuring that they are equipped to effectively manage their role. Retention strategies that target experienced FLNMs are critical to maintaining high levels of performance. Finally, further research exploring the long‐term impact of improvements in the managerial nurse work environment and management experience on performance outcomes in a variety of healthcare settings is needed to guide future interventions and policies.

## About the Statistician

The authors have checked to make sure that our submission conforms as applicable to the Journal's statistical guidelines described here. The statistics were checked before submission by an expert statistician. Statistician's name: Sevan Kutlu His email address: sevankutlu@icloud.com

## Author Contributions

All authors listed meet the authorship criteria according to the latest guidelines of the International Committee of Medical Journal Eda Özlem Ünalda Özlem Ünalditors, and all authors are in agreement with the manuscript. Conceptualization: Tuğba Yeşilyurt Sevim, Eda Özlem Ünalda Özlem Ünalda Özlem ÜnalT., Nilgün Göktepe, Nuray Eda Özlem Ünalda Özlem Ünalrdemir ÇakmakÇ., Eda Özlem Ünalda Özlem Ünalda Özlem ÜnalÖ.Ü., Eda Özlem Ünalda Özlem Ünalsra Adigüzel, Ramiz İşçi; Methodology**:** Tuğba Yeşilyurt Sevim, Eda Özlem Ünalda Özlem Ünalda Özlem ÜnalT., Nilgün Göktepe, Nuray Eda Özlem Ünalda Özlem Ünalrdemir ÇakmakÇ., Eda Özlem Ünalda Özlem Ünalda Özlem ÜnalÖ.Ü., Eda Özlem Ünalda Özlem Ünalsra Adigüzel, Ramiz İşçi; Data collection: Nuray Eda Özlem Ünalda Özlem Ünalrdemir ÇakmakÇ., Eda Özlem Ünalda Özlem Ünalda Özlem ÜnalÖ.Ü., Eda Özlem Ünalda Özlem Ünalsra Adigüzel, Ramiz İşçi; Supervision: Tuğba Yeşilyurt Sevim, Eda Özlem Ünalda Özlem Ünalda Özlem ÜnalT., Nilgün Göktepe; Analysis and/or Interpretation: Eda Özlem Ünalda Özlem Ünalda Özlem ÜnalT.; Literature Search: Tuğba Yeşilyurt Sevim, Eda Özlem Ünalda Özlem Ünalda Özlem ÜnalT., Nilgün Göktepe; Writing Manuscript: Tuğba Yeşilyurt Sevim, Eda Özlem Ünalda Özlem Ünalda Özlem ÜnalT., Nilgün Göktepe; Critical Review: Tuğba Yeşilyurt Sevim, Eda Özlem Ünalda Özlem Ünalda Özlem ÜnalT., Nilgün Göktepe, Nuray Eda Özlem Ünalda Özlem Ünalrdemir ÇakmakÇ., Eda Özlem Ünalda Özlem Ünalda Özlem ÜnalÖ.Ü., Eda Özlem Ünalda Özlem Ünalsra Adigüzel, Ramiz İşçi.

**Permissions:** Institutional permission was received from the administration of the hospital where the research was conducted. Written informed consent was obtained from the nurses who participated in this study.

## Ethics Statement

This study was conducted according to the guidelines of the Declaration of Helsinki, and all procedures involving human subjects were approved by the İstinye University Human Research Ethics Committee (Decision number and date: 22‐19/February 22, 2022). Institutional permission was received from the administration of the hospital where the research was conducted. Written informed consent was obtained from the nurses who participated in this study.

## Conflicts of Interest

The authors declare no conflicts of interest.

## Data Availability

The data that support the findings of this study are available from the corresponding author upon reasonable request.
